# Virtual reality vs. Kalinox® for management of pain in intensive care unit after cardiac surgery: a randomized study

**DOI:** 10.1186/s13613-021-00866-w

**Published:** 2021-05-13

**Authors:** Driss Laghlam, Cecile Naudin, Lucas Coroyer, Vincent Aidan, Julien Malvy, Ghilas Rahoual, Philippe Estagnasié, Pierre Squara

**Affiliations:** 1 Department of Cardiology and Critical Care, CERIC, Clinique Ambroise Paré, 27 boulevard Victor Hugo, 92200 Neuilly-sur-Seine, France; 2 Department of Clinical Research, Clinique Ambroise Paré, 27 boulevard Victor Hugo, 92200 Neuilly-sur-Seine, France

**Keywords:** Analgesia, Perioperative pain, Cardiac surgery, Kalinox®, Virtual reality

## Abstract

**Introduction:**

The management of pain and anxiety remains a challenge in the intensive care unit. By distracting patients, virtual reality (VR) may have a role in painful procedures. We compared VR vs. an inhaled equimolar mixture of N_2_O and O_2_ (Kalinox®) for pain and anxiety management during the removal of chest drains after cardiac surgery.

**Methods:**

Prospective, non-inferiority, open-label study. Patients were randomized, for Kalinox® or VR session during drain removal. The analgesia/nociception index (ANI) was monitored during the procedure for objective assessment of pain and anxiety. The primary endpoint was the ΔANI (ANI_min_ − ANI_0_) during the procedure, based on ANIm (average on 4 min). We prespecified VR as non-inferior to Kalinox® with a margin of 3 points. Self-reported pain and anxiety were also analysed using numeric rate scale (NRS).

**Results:**

200 patients were included, 99 in the VR group and 101 in the Kalinox® group; 90 patients were analysed in both groups in per-protocol analysis. The median age was 68.0 years [60.0–74.8]. The ΔANI was − 15.1 ± 12.9 in the Kalinox® group and − 15.7 ± 11.6 in the VR group (NS). The mean difference was, therefore, − 0.6 [− 3.6 to 2.4], including the non-inferiority margin of 3. Patients in the VR group had a significantly higher pain NRS scale immediately after the drain removal, 5.0 [3.0–7.0] vs. 3.0 [2.0–6.0], *p* = 0.009, but no difference 10 min after. NRS of anxiety did not differ between the two groups.

**Conclusion:**

Based on the ANI, the current study showed that VR did not reach the statistical requirements for a proven non-inferiority vs. Kalinox® in managing pain and anxiety during chest drain removal. Moreover, VR was less effective based on NRS. More studies are needed to determine if VR might have a place in the overall approach to pain and anxiety in intensive care units.

*Trial registration* NCT, NCT03956264. Registered 20 May 2019, https://clinicaltrials.gov/ct2/show/NCT03956264

## Background

The management of acute pain and anxiety related to healthcare interventions remains a major challenge, especially in the intensive care unit (ICU). Pain is common in critically ill adults at rest and during procedures including regular activities (e.g., turning) and discrete procedures (e.g., arterial catheter insertion). Chest tube removal, wound drain removal, and arterial line insertion were described to be the three most painful procedures, with median pain scores of 5 (3–7), 4.5 (2–7), and 4 (2–6) on a 0–10 numeric rating scale, respectively [1].

Following cardiac surgery, mediastinal and pleural chest tubes are inserted to drain blood, then removed typically during the second postoperative day. This withdrawal is described by patients and nurses as a painful and frightening experience [2], and a source of anxiety, especially with regard to fear of pain. After cardiac surgery, 1 week after discharge from ICU, 82% of patients reported pain as the most common traumatic memory of their ICU stay and 6 months later, 38% still recalled pain as their most traumatic ICU memory [3]. Many treatments have been proposed, including analgesics, morphinics, nonsteroidal anti-inflammatory drugs, inhaled nitrous oxide and subcutaneous infiltration of local anaesthetics, such as lidocaine or bupivacaine [4–6]. However, the absence of any treatment is also frequent, given the brevity of the procedure.

An equimolar mixture of oxygen and nitrous oxide (Kalinox®) has been proposed in the management of many anxiogenic and painful situations and was effective in providing analgesia [7–9]. It has been proposed for the ablation of mediastinal redons after cardiac surgery [10].

Virtual reality (VR) is a recent technology that allows the representation of a pleasant environment in three dimensions with complete immersion for the patient, using a helmet. The video quality achieved by this technology and its growing accessibility have attracted the medical community to integrate it into the therapeutic arsenal available to improve the patients’ satisfaction. By distracting patients, this technology helps to reduce anxiety, discomfort and, ultimately, painful feelings related to care [11]. A recent meta-analysis of 20 randomized studies showed a beneficial effect of VR, with a 50% reduction in pain scores [12]. These data suggest that VR might have a role in acutely painful procedures. However, current available studies are clinically and statistically heterogeneous.

The most common scale used for pain or/and anxiety in conscious patients in the ICU is the numeric rating scale (NRS), which is a self-reported scale of feeling, graduated from 0 to 10. This scale is simple, widely used, and easily understood by patients. Analgesic protocols were created to titrate the treatment according to the patient felt pain [13]. In awake patients, current guidelines for prevention and management of pain in ICU recommend the use of self-report scale for pain assessment. Guidelines also suggest that other technology including these measuring HR variabilities, may be of interest in the ICU pain assessment process and should be explored. Although NRS still remains the recommended method in patients able to communicate, electrophysiological tools, not based on patient’s feeling, have been developed to evaluate pain [14].

The analgesia/nociception index (ANI) monitor is a device that collects continuously the patient’s electrocardiogram signal from the scope. The ANI value is based on the influence of the respiratory cycle on the RR interval. It allows a measurement of the heart rate variability, modulated by the parasympathetic nervous system and the sympathetic central nervous system at the sinoatrial node [15]. The ANI monitor gives three values of ANI. A continuous index is displayed (each basic measurement is performed on 64 s of data with a sliding window every second); then, a calculation is made every second and averaged over two time periods: a short average ANIi (average over 2 min) and a longer average ANIm (average over 4 min).

A mathematic analysis is made by the monitor to normalize the ANI values between 0 (maximum sympathetic effect, indicating the highest level of stress) and 100 (maximum parasympathetic effect, indicating a low level of stress) [16]. In other words, higher ANI values would typically be associated with lower pain scores. It has been demonstrated that the ANI is useful to guide analgesic titration during surgery [17], to evaluate postoperative pain [18] and emotional status [19]. When used in routine care procedures in critically ill non-comatose patients, ANI values were significantly correlated with Behavioral Pain Scales and instant ANI ≥ 43 had a negative-predictive value of 90% for excluding significant pain [20]. In another study, in the immediate postoperative context in conscient patients but before tracheal extubation, the sensitivity and specificity of ANI < 50 to discriminate between patients with NRS ≤ 3 and NRS > 3 were both 86%, giving a 92% negative predictive value, and an area under the ROC curve of 0.89 [18].

This study aimed to compare VR vs. Kalinox® for pain and anxiety management during the removal of chest drains after cardiac surgery, based on ANI values.

## Materials and methods

### Design, patients and randomization

This prospective, comparative, non-inferiority, open label and randomized trial was conducted in the ICU of the Centre Médico-Chirurgical Ambroise Paré, in Neuilly-sur-Seine, France. All patients underwent cardiac surgery, requiring withdrawal of mediastinal and pleural drains. The patients were included, after information and signed informed consent, if they were over 18 years, extubated and in sinus rhythm on the electrocardiogram at the time of the protocol, since ANI signal analysis requires a sinus rhythm. Exclusion criteria included: patients with a pacemaker, contraindication to Kalinox® or/and morphine, altered visual acuity preventing use of VR, incapacity to understand the protocol, and patients under protection of adults (guardianship, curator or safeguard of justice). Randomization was performed using an external Interactive Web Response System. Patients were randomly assigned (1:1), in permuted blocks of six, to have either hypnotic treatment with Kalinox® or a session of VR during the removal of drain. The trial was registered on clinicaltrials.gov (NCT03956264) on May 20, 2019. Oversight and study approval were provided by the Committee for Protection of Human Subjects (CPP SUD-EST VI-AU 1500) on March 8, 2019.

### Protocol

The removal of mediastinal and pleural drains was considered from the second day after cardiac surgery, following the specific protocol of the unit, if the drain did not produce over 100 cc per day and if there was no medical contraindication, such as pneumothorax, or suspicion of mediastinal or pleural infection. The withdrawal was performed by the nurse on medical prescription and could be done in the ICU or in the standard care unit. The ANI system (V2, Software version: V2.2.1.0., PhysioDoloris®, MDoloris Medical Systems, Loos, France), with specific precordium electrodes connected to the monitor, was installed 15 min before the procedure to ascertain a good and stable signal. In practice, the drain removal started with a meticulous disinfection of the sternal scar and redon orifice, followed by section of the sutures. This preparative time was immediately followed by a removal of the drains, one by one. In the two groups, nefopam (120 mg per day in continuous infusion, in the absence of contraindication) was systematically administered as a pre-procedural analgesic treatment. Morphine could be used in an intravenous titration at any time, in the event of moderate to severe pain (NRS > 4). Continuous ANI data were extracted after the procedure. We recorded the values of ANIm (average over 4 min). The baseline ANI before drain removal (ANI_0_) and the minimal value reached during the procedure (ANI_min_) were used to derive the ANI variation (ΔANI), as ANI_min_ − ANI_0_, and the NRS of pain and anxiety was recorded before the drain removal, immediately after, and 10 min after the procedure. We also reported the amount of morphine used and the satisfaction of the patient and nurses after the procedure using a four-level rating scale (very satisfied, satisfied, poorly satisfied, unsatisfied).

The two specific protocols for the two groups of the study were as follows:

- Group 1: A test session of VR was performed after randomization and before the procedure.
  Establishment of a communication code before starting the VR was performed to warned patients before removing the tube (e.g., gentle touch of the arm). The VR session started during the preparative phase, at least 5 min before the removal of the drains, and was continued for 10 min after. We used a VRx helmet 90-degree field of view with head tracking, [Deepsen, DT Didier, Mont d’Or, France (http://www.deepsen.io/)]. Patients had a choice between five different immersive environments (360° videos): a snowy mountain, a landscape in India or in Camargue (France), a balloon ride or a canoe descent.
- Group 1: Kalinox® (Air Liquide, Paris France) was started 1 min before the removal of the drains, delivered continuously and stopped 1 min after removal to avoid side effects. Patients were warned orally before drains removal.

### Endpoint

The primary endpoint was the objective assessment of pain and anxiety, measured by the ΔANI during the chest drain removal, to demonstrate that VR is non-inferior to Kalinox®. Secondary outcomes were the duration of ANI > 60, self-reported NRS ratings for pain and anxiety (before, immediately after and 10 min after the procedure), the use of morphine (Y/N), patient and nurse satisfaction (four-level Likert scale) and side effects, such as digestive or/and neurological disorders.

### Statistical analysis

We initially calculated a total sample size of 176 participants to obtain 80% power to show a non-inferior ΔANI in the VR group compared with the Kalinox® group, at a 5% significance level [21]. The ΔANI in the VR group was prespecified as non-inferior to that of the Kalinox® group if the difference was 3 points or less. We initially assumed a normally distributed ΔANI of 25 ± 8 in the 2 groups and a maximum of 10% of attrition. The total number of patients was planned to be 100 patients per group.

Shapiro tests were used to test the normality of distribution of the studied variables. Continuous variables were expressed as mean ± standard deviation (SD) when distributed normally and median [interquartile range] when not. Categorical variables were expressed as a number (percentage). Categorical variables in two groups were compared using the Chi-squared and Pearson’s tests. Comparison of normally distributed variables used the Student-*t* test, and other continuous variables were compared by a Mann–Whitney nonparametric test. The software used was SPSS 25.0.

## Results

**Fig. 1:**
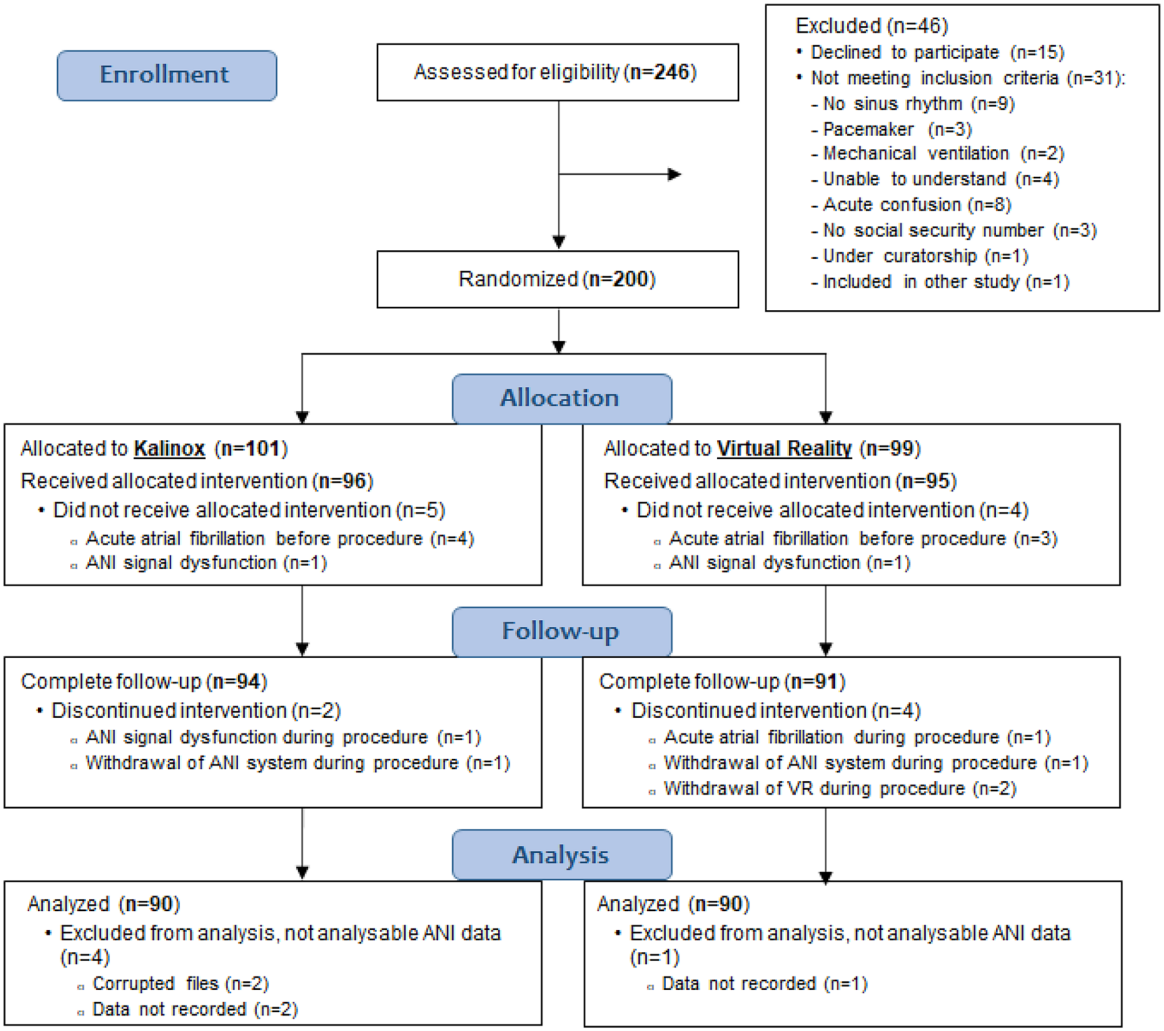
Flow chart

From September 2019 to July 2020, 246 adult patients were screened. Of those, 15 refused to participate in the study and 31 did not meet the inclusion criteria. Finally, 200 patients gave written informed consent and were randomized to the VR group (*n* = 99) or to the Kalinox® group (*n* = 101), 96 patients received the treatment and 90 were analysed in per protocol analysis (11 excluded patients: 4 with acute atrial fibrillation before protocol, 7 with non-analysable ANI data). In the VR group, 95 patients received the VR session and 90 were analysed (9 excluded patients: 4 atrial fibrillation before/during protocol, 3 non analysable ANI data, and 2 withdrew the helmet during the procedure). A flow diagram is presented in Fig. 1.

Patient baseline characteristics are presented in Table 1. The median age of the whole cohort was 68.0 [60.0–74.8] years. Gender did not significantly differ between the two groups: 62 men (69%) in the VR group vs. 72 (80%) in the Kalinox® group; *p* = 0.09. The types of surgery were similar in the two groups.

**Table 1:** Baseline characteristics

	Whole Cohort (*n* = 180)	Kalinox® (*n* = 90)	VR (*n* = 90)	*p* value
Demographics				
Male gender	134 (74)	72 (80)	62 (69)	0.09
Age, years	68.0 [60.0–74.8]	68.0 [60.0–74.0]	68.0 [61.5–75.0]	0.94
BMI, kg/m^2^	25.9 [23.5–28.7]	26.5 [23.7–29.3]	25.1 [23.2–28.1]	0.17
Surgery				
CPB time	70 [55–86]	71 [55–85]	69 [55–87]	0.74
Type of surgery				0.39
Isolated coronary bypass surgery	99 (55.0)	53 (58.9)	46 (51.1)	
Combined coronary bypass surgery	7 (3.9)	5 (5.6)	2 (2.2)	
Isolated or combined valve surgery	61 (33.9)	29 (32.2)	32 (35.6)	
Aorta surgery	5 (2.8)	1 (1.1)	4 (4.4)	
Myxoma	1 (0.6)	0 (0.0)	1 (1.1)	
Pericardiocentesis for tamponade	6 (3.3)	2 (2.2)	4 (4.5)	
Abdominal aorta surgery	1 (0.6)	0 (0.0)	1 (1.1)	
Number of mediastinal tubes	2 [2–2]	2 [2–2]	2 [2–2]	0.14
Number of pleural tubes	1 [0–2]	1 [0–2]	1 [0–2]	0.43
Drains removal in ICU	175 (97.2)	88 (97.8)	87 (96.7)	1.0
Drains removal in standard care unit	5 (2.8)	2 (2.2)	3 (3.3)	1.0

Values are given in median [25–75 interquartile range] or number (%)

*BMI* body mass index, *CPB* cardiopulmonary bypass, *ICU* intensive care unit, *VR* virtual reality

**Fig. 2:**
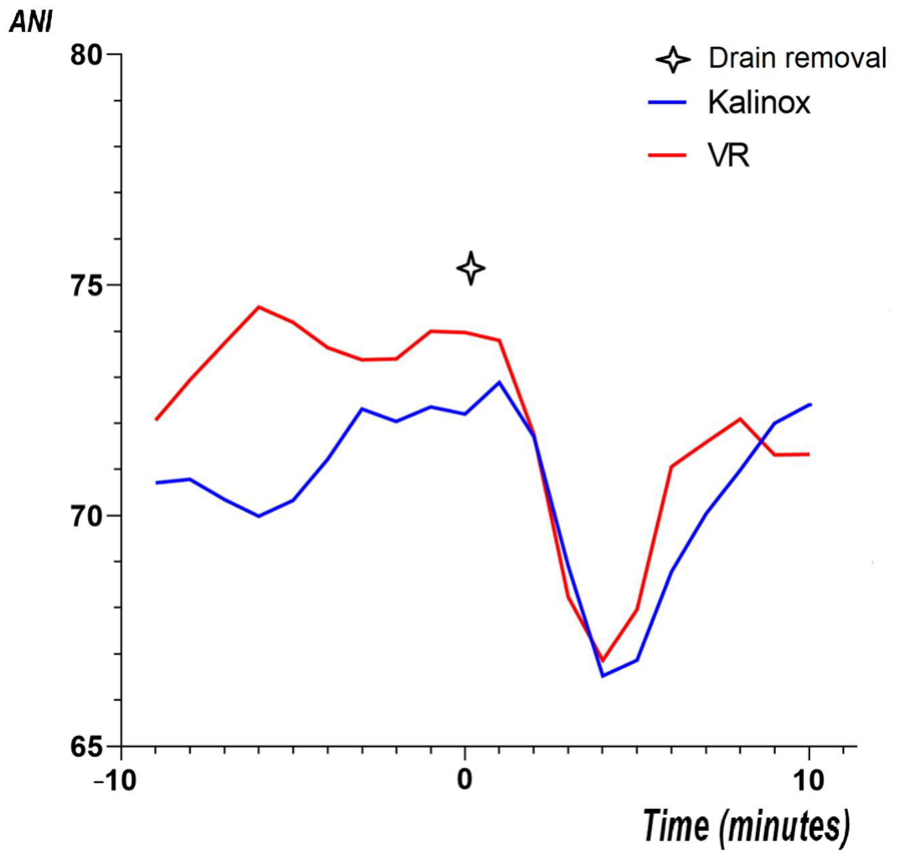
ANI variation during procedure. ANI values 0–100 points (low values indicating higher levels of pain). *ANI* analgesia/nociception index, *VR* virtual reality

**Fig. 3:**
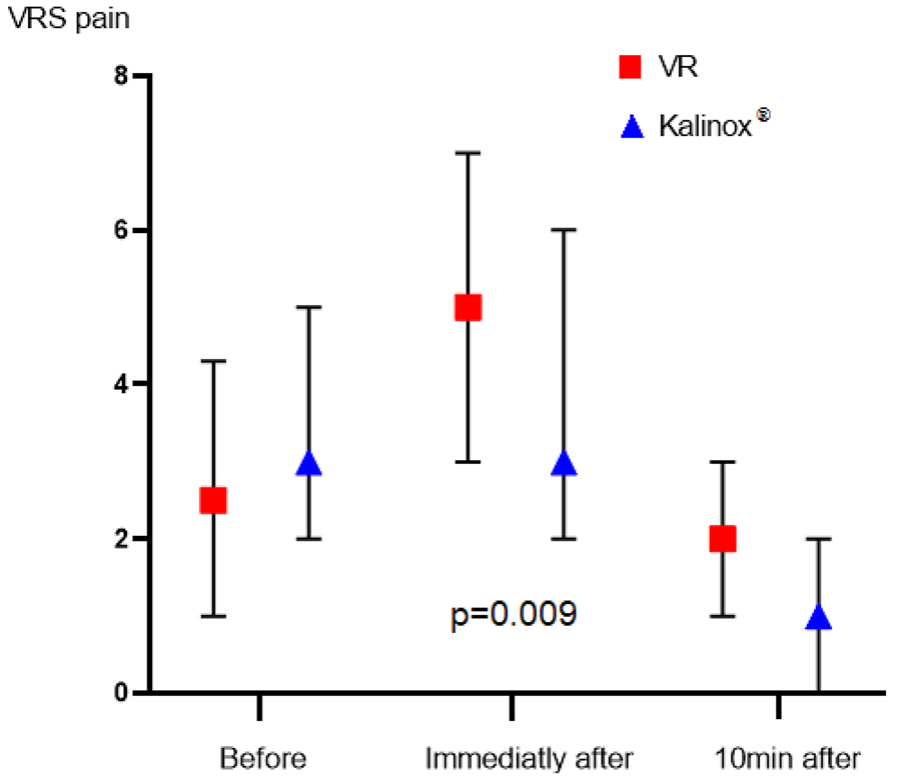
NRS pain variation during procedure. NRS pain (0–10) in the VR and Kalinox groups before, immediately after, and 10 min after chest drain removal. Data are represented as median with interquartile range. *NRS* numerical rating scale, *VR* virtual reality

In the primary analysis, the ΔANI was − 15.7 ± 11.6 in the VR group and − 15.1 ± 12.9 in the Kalinox® group; the mean difference was, therefore, − 0.6, with a 95% confidence interval of − 3.6 to 2.4. The lower limit of the 95% CI for the reported difference was, therefore, lower than the non-inferiority margin (3.6 vs. 3). The ANI data and trial outcomes are presented in Table 2. Variations of ANI during the procedure are reported in Fig. 2. The percentage of time spent within the recommended range of the ANI (ANI > 60) was 85 [44–100] in the VR vs. 77 [39–100] in the Kalinox® group, *p* = 0.503. The number of patients having an ANI below two different thresholds of 40 and 50 during the procedure was not significantly different between the two groups. In contrast, patients in the VR group had significantly more pain based on the NRS scale, immediately after the drain removal compared to patients in the Kalinox® group, 5.0 [3.0–7.0] vs. 3.0 [2.0–6.0], respectively, *p* = 0.009, but there was no difference in the pain level 10 min after the procedure. Variations of NRS pain during the procedure are presented in Fig. 3. The NRS anxiety level did not differ between the two groups. The use of morphine was low in the two hours before the procedure (7.2% in the whole cohort) and was not significantly different between the two groups during the procedure: 2 (2.2%) in the VR group vs. 0 (0%) in the Kalinox® group (*p* = 0.155). Patients’ satisfaction was higher in the Kalinox® group (*p* = 0.002). In detail, 89 (99%) patients were satisfied and very satisfied with Kalinox® vs. 81 (90%) with VR, *p* = 0.009. The nurses’ satisfaction (satisfied and very satisfied) was 97.2% in the whole cohort and did not differ between the two groups (*p* = 0.69). Three minor side effects were declared in the VR group (2 vertigo and 1 nausea) and also three in the Kalinox® group (2 euphoria and 1 headache).

**Table 2:** Outcomes

	Whole cohort (*n* = 180)	Kalinox® (*n* = 90)	VR (*n* = 90)	*p* value	
				PP	ITT
Primary outcome					
ΔANI	− 15.38 ± 12.20	− 15.06 ± 12.85	− 15.70 ± 11.58		
Secondary outcomes					
Time with ANI > 60 (%)	83 [43–100]	77 [39–100]	85 [44–100]	0.503	
ANI minimal value, *n* (%):					
ANI < 50	56 (31.1)	30 (33.3)	26 (28.9)	0.63	
ANI < 40	16 (8.9)	9 (10)	7 (7.8)	0.79	
NRS pain					
Before procedure	3.0 [1.0–5.0]	3.0 [2.0–5.0]	2.5 [1.0–4.3]	0.164	0.29
Immediately after procedure	4.0 [2.0–6.0]	3.0 [2.0–6.0]	5.0 [3.0–7.0]	0.009	0.03
10 min after procedure	2.0 [1.0–3.0]	1.0 [0.0–2.0]	2.0 [1.0–3.0]	0.065	0.06
NRS anxiety					
Before procedure	3.0 [1.0–5.0]	4.0 [1.5–5.0]	3.0 [1.0–5.0]	0.407	
Immediately after procedure	3.0 [1.0–5.0]	3.0 [1.0–5.0]	3.0 [1.0–6.0]	0.444	
10 min after procedure	1.0 [0.0–3.0]	1.0 [0.0–3.0]	1.0 [0.0–3.0]	0.785	
Morphine use					
Before procedure	13 (7.2)	7 (7.8)	6 (6.7)	0.773	
After procedure	2 (1.1)	0 (0.0)	2 (2.2)	0.155	
Patient satisfaction				0.002	
Unsatisfied	0 (0)	0 (0)	0 (0)	–	
Not very satisfied	10 (5.6)	1 (1.1)	9 (10)	0.01	
Satisfied	58 (32.2)	23 (25.6)	35 (38.9)	0.06	
Very satisfied	112 (62.2)	66 (73.3)	46 (51.1)	0.002	
Nurse satisfaction				0.69	
Unsatisfied	0 (0)	0 (0)	0 (0)	–	
Poorly satisfied	5 (2.8)	2 (2.2)	3 (3.3)	0.65	
Satisfied	21 (11.6)	9 (10)	12 (13.3)	0.49	
Very satisfied	154 (85.6)	79 (87.8)	75 (83.3)	0.40	

Values are given in mean ± SD when appropriate or median [25–75 interquartile range] or n (%)

*ANI* analgesia/nociception index 0–100, *ITT* intention to treat analysis, *NRS* numerical rating scale 0–10, *PP* per-protocol analysis, *VR* virtual reality

## Discussion

Although easy to use, well tolerated and providing satisfaction in 90% of patients and 97% of nurses, our study failed to prove the non-inferiority of VR compared to Kalinox® when removing drains after cardiac surgery. Based on the ANI, the results were not statistically different; however, they did not reach the statistical requirements for a proven non-inferiority. Based on the NRS and on patient satisfaction, the results were significantly in favour of Kalinox®: the feeling of pain immediately after the procedure in the VR group was significantly higher than in the Kalinox® group. This is at odds with the results of a recent meta-analysis, where VR was able to reduce pain in various painful situations, such as burn wound care. However, these results were based on small studies that were clinically and statistically heterogeneous, using only a pain score [12]. Some previous reviews and studies found moderate evidence for the reduction of acute pain with VR, based on self-rated NRS [22–24]. In this current study, we did not find a correlation between ANI values and NRS, as ANI did not differ between the two groups, while NRS was significantly higher in VR group. One hypothesis is that the difference could be due to a sedative and/or amnesic effect of Kalinox®.

Pain management in the ICU is still a challenge, especially in cases of brief painful care, such as during drain removal. Opioid drug use is not the best treatment option in this situation due to their onset and duration of action and their digestive and neurological side effects. Besides, morphine use was extremely low in this study. Kalinox® seems to be a good compromise, because it is easy to use, has a short onset of action and a short duration after removal. Moreover, it has shown a reduction of acute pain and anxiety with low side effects.

Anxiety is also an important issue in the ICU and could result in persistent symptoms after discharge and/or depression [25]. In addition, perioperative depression and anxiety may be associated with increased postoperative mortality in patients undergoing cardiac surgery [26, 27]. In our study, we found no difference between VR and Kalinox® in terms of self-reported anxiety immediately after the procedure and after 10 min. Using VR to distract patients could be an efficient tool to reduce anxiety, notably during painful procedures in the ICU. VR must have special qualities to make it effective, such as presence (a sense of immersion in the environment), interactivity, customization, social interaction, and embodiment. This allows it to be accepted by the patient and incorporated successfully into their existing medical therapies [28]. Although these explanations were developed from the observation of children, they are comparable for adults. In our study, despite a median age of 68 [60–74.8] years, VR was well tolerated and accepted (90% of patients were satisfied or very satisfied). To increase the acceptance, we performed a test session of VR before the procedure.

Although limited interest to reduce pain, VR seems to be a promising adjunctive therapy in ICU, as it is well-tolerated, non-invasive, and could reduce stress and anxiety. The improvement of VR devices with higher 360 video quality, more immersive environments with the possibility of adding hypnosis techniques, as well as the greater availability of this type of accessory in ICU, makes it an interesting tool to experiment in near future.

This study presented some limitations. First, it was a single-centre study, corresponding to a specific experience. We performed this study on a specific situation (chest tube removal) in cardiac surgery patients, which can make it difficult to generalize. Patients were not included if they were judged unable to understand by investigators, but we did not use delirium evaluation with specific scale. Second, we used a specific VRx device, and each VR device may not be suitable for all patients. With this device, patients could choose their immersive environment and navigate into it. Some patients may need more interactivity, self-customization and social interaction for a full VR experience, and this could have reduced the effectiveness of the VR session. Furthermore, we fixed a range of non-inferiority of 3 and anticipated a change of ΔANI of 25 ± 8. This threshold could have been too restrictive. Finally, we could not exclude a lack of power to demonstrate the non-inferiority of VR and Kalinox®.

## Conclusion

Although VR was well tolerated by patients and allowed a satisfying self-reported anxiety control during drain removal after cardiac surgery, it failed to prove non-inferiority compared to Kalinox® for the management of pain and anxiety, as assessed by ANI, and was less effective based on NRS. More studies are needed to determine if VR might have a place in the overall approach to pain and anxiety in intensive care units.

## Data Availability

The data sets used and analysed during the current study are available from the corresponding author on reasonable request.

## Abbreviations

ANI: Analgesia/nociception index
ICU: Intensive care unit
NRS: Numerical rating scale
SD: Standard deviation
VR: Virtual reality

## References

[1] Puntillo KA, Max A, Timsit JF, Vignoud L, Chanques G, Robleda G (2014). Determinants of procedural pain intensity in the intensive care unit. The Europain® study. Am J Respir Crit Care Med.

[2] Gift AG, Bolgiano CS, Cunningham J (1991). Sensations during chest tube removal. Heart Lung.

[3] Schelling G, Richter M, Roozendaal B, Rothenhäusler HB, Krauseneck T, Stoll C (2003). Exposure to high stress in the intensive care unit may have negative effects on health-related quality-of-life outcomes after cardiac surgery. Crit Care Med.

[4] Carson MM, Barton DM, Morrison CC, Tribble CG (1994). Managing pain during mediastinal chest tube removal. Heart Lung.

[5] Bruce EA, Howard RF, Franck LS (2006). Chest drain removal pain and its management: a literature review. J Clin Nurs.

[6] Akrofi M, Miller S, Colfar S, Corry PR, Fabri BM, Pullan MD (2005). A randomized comparison of three methods of analgesia for chest drain removal in postcardiac surgical patients. Anesth Analg.

[7] Brotzman EA, Sandoval LF, Crane J (2018). Use of nitrous oxide in dermatology: a systematic review. Dermatol Surg.

[8] Buhre W, Disma N, Hendrickx J, DeHert S, Hollmann MW, Huhn R (2019). European society of anaesthesiology task force on nitrous oxide: a narrative review of its role in clinical practice. Br J Anaesth.

[9] Gao LL, Yu JQ, Liu Q, Gao HX, Dai YL, Zhang JJ (2019). Analgesic effect of nitrous oxide/oxygen mixture for traumatic pain in the emergency department: a randomized, double-blind study. J Emerg Med.

[10] Thompson JM, Neave N, Moss MC, Scholey AB, Wesnes K, Girdler NM (1999). Comparison of Entonox and low-dose premixed isoflurane and desflurane for chest drain removal after cardiac surgery. Br J Anaesth.

[11] Pourmand A, Davis S, Lee D, Barber S, Sikka N (2017). Emerging utility of virtual reality as a multidisciplinary tool in clinical medicine. Games Health J.

[12] Chan E, Foster S, Sambell R, Leong P (2018). Clinical efficacy of virtual reality for acute procedural pain management: a systematic review and meta-analysis. PLoS ONE.

[13] Safikhani S, Gries KS, Trudeau JJ, Reasner D, Rüdell K, Coons SJ (2017). Response scale selection in adult pain measures: results from a literature review. J Patient Rep Outcomes.

[14] Devlin JW, Skrobik Y, Gélinas C, Needham DM, Slooter AJC, Pandharipande PP (2018). Executive summary: clinical practice guidelines for the prevention and management of pain, agitation/sedation, delirium, immobility, and sleep disruption in adult patients in the ICU. Crit Care Med.

[15] Logier R, Jeanne M, De Jonckheere J, Dassonneville A, Delecroix M, Tavernier B. PhysioDoloris: a monitoring device for analgesia/nociception balance evaluation using heart rate variability analysis. In: 2010 annual international conference of the IEEE engineering in medicine and biology. 2010. p. 1194–7. 10.1109/IEMBS.2010.5625971 21095676

[16] Pichot V, Gaspoz JM, Molliex S, Antoniadis A, Busso T, Roche F (1999). Wavelet transform to quantify heart rate variability and to assess its instantaneous changes. J Appl Physiol.

[17] Theerth KA, Sriganesh K, Reddy KM, Chakrabarti D, Umamaheswara Rao GS (2018). Analgesia Nociception Index-guided intraoperative fentanyl consumption and postoperative analgesia in patients receiving scalp block versus incision-site infiltration for craniotomy. Minerva Anestesiol.

[18] Boselli E, Bouvet L, Bégou G, Dabouz R, Davidson J, Deloste JY (2014). Prediction of immediate postoperative pain using the analgesia/nociception index: a prospective observational study. Br J Anaesth.

[19] Abdullayev R, Yildirim E, Celik B, Topcu Sarica L (2019). Analgesia Nociception Index: heart rate variability analysis of emotional status. Cureus.

[20] Chanques G, Tarri T, Ride A, Prades A, De Jong A, Carr J (2017). Analgesia nociception index for the assessment of pain in critically ill patients: a diagnostic accuracy study. Br J Anaesth.

[21] Benner A. Sample size tables for clinical studies. (2nd edn). David Machin, Michael J. Campbell, Peter M. Fayers and Alain P. Y. Pinol, Blackwell Science Ltd., Oxford, 1997. No. of pages: x+315. Price: £45. ISBN 0-86542-870-0. Statistics in Medicine. 1999;18(4):494–5.

[22] Walker MR, Kallingal GJ, Musser JE, Folen R, Stetz MC, Clark JY (2014). Treatment efficacy of virtual reality distraction in the reduction of pain and anxiety during cystoscopy. Mil Med.

[23] Kipping B, Rodger S, Miller K, Kimble RM (2012). Virtual reality for acute pain reduction in adolescents undergoing burn wound care: a prospective randomized controlled trial. Burns.

[24] Garrett B, Taverner T, Masinde W, Gromala D, Shaw C, Negraeff M (2014). A rapid evidence assessment of immersive virtual reality as an adjunct therapy in acute pain management in clinical practice. Clin J Pain.

[25] Nikayin S, Rabiee A, Hashem MD, Huang M, Bienvenu OJ, Turnbull AE (2016). Anxiety symptoms in survivors of critical illness: a systematic review and meta-analysis. Gen Hosp Psychiatry.

[26] Takagi H, Ando T, Umemoto T (2017). Perioperative depression or anxiety and postoperative mortality in cardiac surgery: a systematic review and meta-analysis. Heart Vessels.

[27] Pochard F, Bellivier F, Faessel AI, Squara P (1997). Anxiety and depressive disorders in cardiovascular diseases. Encephale.

[28] Won AS, Bailey J, Bailenson J, Tataru C, Yoon IA, Golianu B (2017). Immersive virtual reality for pediatric pain. Children.

